# Role and therapeutic targets of P2X7 receptors in neurodegenerative diseases

**DOI:** 10.3389/fimmu.2024.1345625

**Published:** 2024-02-02

**Authors:** Huiyong Zheng, Qiang Liu, Siwei Zhou, Hongliang Luo, Wenjun Zhang

**Affiliations:** ^1^ Second Clinical Medical School, Jiangxi Medical College, Nanchang University, Nanchang, China; ^2^ Gastrointestinal Surgery, The Second Affiliated Hospital, Jiangxi Medical College, Nanchang University, Nanchang, China; ^3^ Department of Rehabilitation Medicine, The Second Affiliated Hospital, Jiangxi Medical College, Nanchang University, Nanchang, China

**Keywords:** P2X7 receptor, neurodegenerative disease, neuroinflammation, antagonist, microglia

## Abstract

The P2X7 receptor (P2X7R), a non-selective cation channel modulated by adenosine triphosphate (ATP), localizes to microglia, astrocytes, oligodendrocytes, and neurons in the central nervous system, with the most incredible abundance in microglia. P2X7R partake in various signaling pathways, engaging in the immune response, the release of neurotransmitters, oxidative stress, cell division, and programmed cell death. When neurodegenerative diseases result in neuronal apoptosis and necrosis, ATP activates the P2X7R. This activation induces the release of biologically active molecules such as pro-inflammatory cytokines, chemokines, proteases, reactive oxygen species, and excitotoxic glutamate/ATP. Subsequently, this leads to neuroinflammation, which exacerbates neuronal involvement. The P2X7R is essential in the development of neurodegenerative diseases. This implies that it has potential as a drug target and could be treated using P2X7R antagonists that are able to cross the blood-brain barrier. This review will comprehensively and objectively discuss recent research breakthroughs on P2X7R genes, their structural features, functional properties, signaling pathways, and their roles in neurodegenerative diseases and possible therapies.

## 1 Introduction

P2X7 is a microglial protein highly expressed in the central nervous system (CNS) and functions as an ATP-gated ion channel (1). It plays a crucial role in mediating ATP-driven hazardous signaling, as its activation leads to the opening of pores and non-selective transport of Ca^2+^, Na^+^, and K^+^ (2). The expression of P2X7R in immunocompetent cells of the central and peripheral nervous system has been extensively described (3). It is expressed in microglia, astrocytes, and oligodendrocytes in the CNS (4). However, there is an ongoing discussion regarding its expression in neurons (5, 6). The P2X7-EFGP BAC transgenic mouse model overexpresses functional fluorescently labeled P2X7, which is located at the protein level and provides stronger signaling. However, neuronal P2X7 protein expression is not induced under pathological conditions (7). The study notes that P2X7 is predominantly found in microglia and oligodendrocytes. Recently, researchers generated humanized P2X7R (hP2RX7) by inserting human P2RX7 cDNA into the mouse P2RX7 locus. They found that P2X7R is specifically expressed in glutamatergic pyramidal neurons in the hippocampus (8). P2X7R expression was also found on neuronal progenitor cells and mature neuronal cells of human hiPSC origin. This study also indicated that P2X7R is not localized to the cell membrane of neurons and may not directly mediate neurotoxicity (9). The presence of P2X7R in neurons and glial cell populations is supported by the P2RX7-EGFP reporter mouse, which expresses enhanced green fluorescent protein (EGFP) (10).

ATP is a co-transmitter released by neurons and can be influx into the extracellular space from glial cells (astrocytes, oligodendrocytes, microglia) in the CNS to regulate neuronal activity (11). P2X7 is extensively expressed in microglial cells within the CNS (12). Activating P2X7 initiates the assembly of NLR family pyrin structural domains containing NLRP3 in microglia. This results in the activation of cysteinyl asparagine-1, which increases cellular metabolism by boosting both glycolysis and oxidative phosphorylation. In turn, this causes the secretion of IL-1β, IL-6, TNF-α, and IL-18, thereby initiating a neuroinflammatory response (13, 14). Stimulation of P2X7 also induces the discharge of different pro-inflammatory substances like TNF-α (15), IL-6 (16), CCL2 (17), excitotoxic glutamate (17), and reactive oxygen species (ROS) (18). These mediators result in neuroinflammation, proliferation of reactive glial cells, and cell death. It is important to note that these substances cause neuroinflammation and cellular damage. The main pathogenic alterations in neurodegenerative disorders, like Alzheimer’s disease (AD), Parkinson’s disease (PD), Huntington’s disease (HD), Multiple sclerosis (MS), and Amyotrophic lateral sclerosis (ALS) that are prevalent globally, consist of several neurodegenerative reactions, leading to substantial ATP release through permeable plasma membranes of neural tissues (19). This results in high ATP concentration activating the P2X7R, causing neurological damage. Therefore, extensive research explores the modulation of P2X7R-mediated pathways as a possible treatment for neurodegenerative diseases, aiming to slow or remedy their progression. Our review concentrates on the P2X7R signaling pathway, the extent of its participation in neurodegenerative disorders, and available therapeutic interventions.

## 2 Overview of purinergic receptor P2X7R

### 2.1 Genes encoding for P2X7R

**Figure 1:**
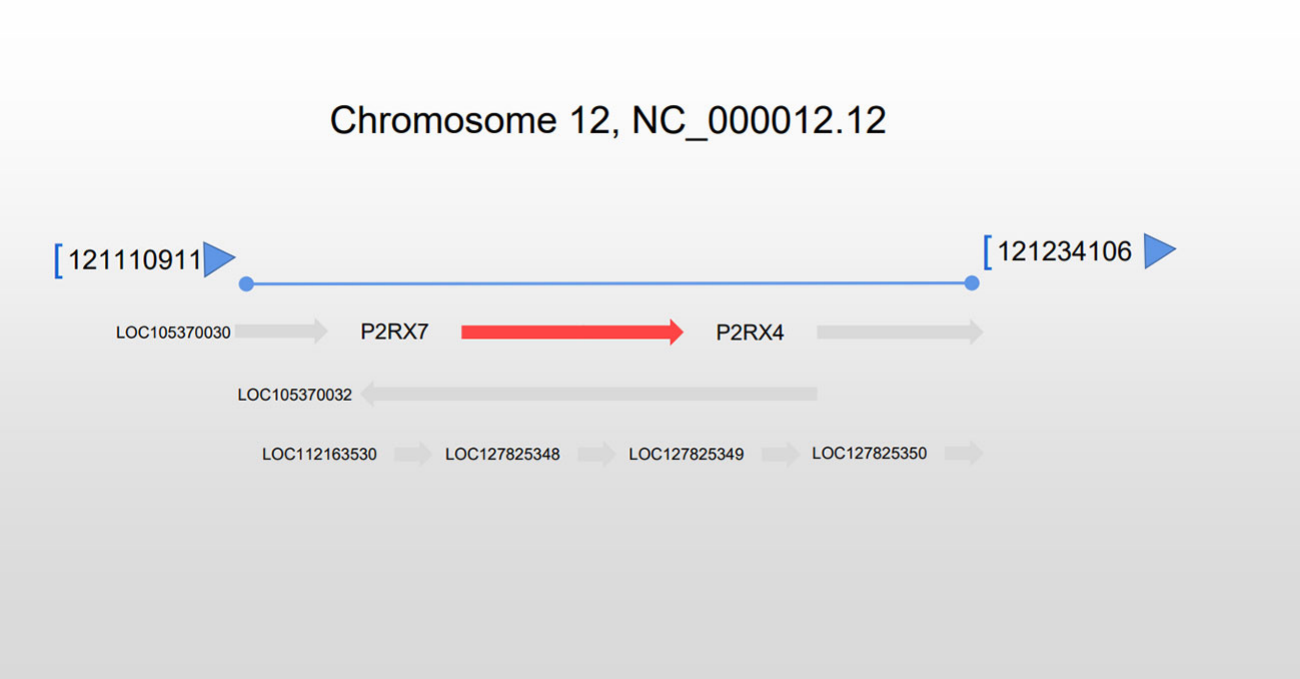
Location of the P2RX7 gene. The P2RX7 gene is situated on chromosome 12 at q24.31, extending 53 kb, and situated next to the P2RX4 gene.

The P2X7 gene, which codes for the P2X7R, is situated on the long arm of chromosome 12 at 12q24.31 with a length of 53 kb along with 13 exons (**Figure 1**). P2RX4 (12.q24.32) is located in proximity to a mitophagosome. (Source: www.ncbi.nlm.nih.gov/gene/5027).

### 2.2 Structure of the P2X7R

P2XR is a trimeric ion channel composed of three subunits. Each subunit contains two structural domains: the extracellular cysteine-rich structural domain and the C- and N-termini that enhance channel function (20). P2X7R belongs to the P2XR family and is extensively expressed in body cells, particularly in macrophages and microglia (21). Furthermore, the P2X7R has ATP-gated ion channel activity (22). The P2X7R requires higher levels of ATP to activate compared to other P2XR (23). P2X7 encodes a 595-amino acid protein, which forms a trimeric ion channel assembly (23). The P2X7R protein comprises cellular extracellular, transmembrane, and cytoplasmic components (24). The ligand binding site is formed through subunit interactions among three extracellular structural domains, totaling 282 amino acids (13, 25). Six alpha subunits form two 24 amino acid transmembrane helices, consisting of three TM1 and three TM2 units (26). The cytoplasmic cap is composed of two N-terminal β strands, which converge proximally at the C-terminus with β15 and α8 (25). The N-terminus starts about 26 amino acids earlier and includes a conserved protein kinase C (PKC) phosphorylation consensus site [TX (K/R)] (13). Following this, TM1 is the first transmembrane structural domain that ranges from a26 to 46 and contains the ATP-binding pocket (27). It is then followed by the voluminous extracellular structural domain that maintains conformational stability through a highly conserved protein fold constructed with multiple disulfide bonds between cysteine residues (28). The second transmembrane structural domain following the extracellular area is TM2 (amino acid 330 to 349), which contains numerous essential pore-lining residues (28). These residues regulate channel gating: S342 is situated at the narrowest segment of the channel, and Y343 is phosphorylated to modify gating (29).

The P2X7R has the lengthiest C-terminal among all P2XR (30). Moreover, in the P2X7 subunit, the carboxyl-terminal tail (amino acid 356-595) is the most remarkable structural domain, stabilizing macropore opening, distinct to this receptor subtype. The initial region of the C-terminal tail has a cysteine-rich area and is palmitoylated on at least five residues, specifically C362, C363, C374, C377, and S360. The palmitoylated residues serve as hinges, thus enabling each C-terminal tail to form a binding site for guanosine diphosphate or triphosphate (GDP/GTP) and two zinc bins (31).

**Figure 2:**
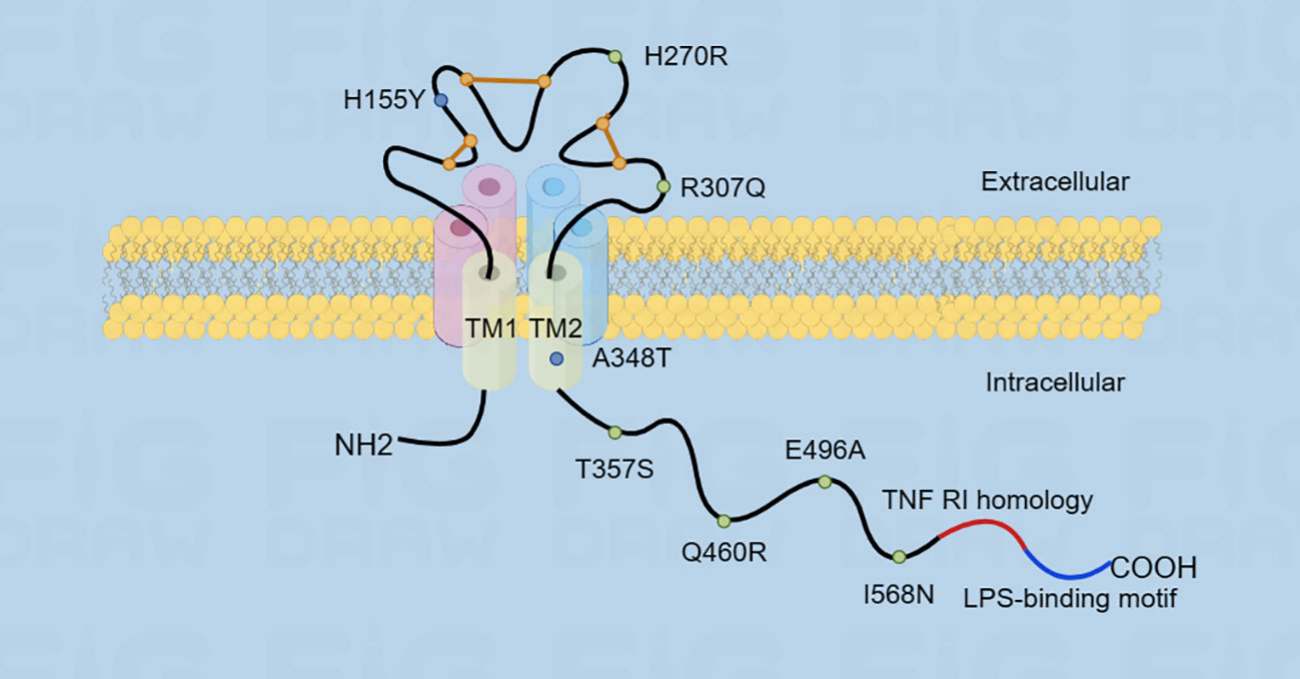
The figure illustrates the structure of P2X7R, which consists of transmembrane structural domains TM1 and TM2. The extracellular structural domain is represented by an orange line, which maintains the conformational stability through a disulfide bond. The carboxyl-terminal tail is very long and contains two homologous region sequences. Additionally, the figure shows the location of single nucleotide polymorphisms (SNPs) in the P2X7 receptor, with gain-of-function SNPs represented by blue dots and loss-of-function SNPs represented by green dots.

Multiple motifs that bind lipids and proteins are present in this structural domain. Specifically, the region 436-531 shares homology with a segment of tumor necrosis factor receptor 1 (TNFR1) that includes the death domain, while residues 573-590 exhibit homology with the endotoxin- binding region of the serum LPS-binding protein (LBP). Additionally, there are multiple regions that share homology (32) (**Figure 2**).

### 2.3 Features of P2X7R

The P2X7R is notable within the P2XR family for its distinct characteristics. Structurally, it consists of 3-6 homologous subunits (33) that commonly combine to form a trimeric complex in order to create a functional P2X7R. The C-terminus of the P2X7R is lengthier compared to that of other P2X7R. It plays a role in regulating the receptor’s functions, which include signaling pathways, cellular localization, protein-protein interactions, and post-translational modifications (34, 35). As an ATP-gated non-selective cation channel, the P2X7R mediates the inward flow of Na^+^ and Ca^2+^ and the efflux of K^+^, resulting in inward current/depolarization (36, 37). Normal physiological conditions are maintained by extracellular divalent cations such as calcium, magnesium, zinc, and copper ions (38–40), protons (41), and anions (42), which keep receptor activity at low levels. There are two potential reasons for the expansion of P2X7R. One is its binding to the agonist-binding pocket over an extended period, which causes the channel to expand gradually (43). The other is that P2X7R is able to transport large organic cations directly (44) or form a large conductance pore (45). P2X7R has a different conductance than other P2XRs in terms of its response to activation. In comparison, other receptors show a fast and brief response that diminishes within a few seconds. On the other hand, P2X7R do not typically exhibit desensitization and allow for continued inward flow of Ca^2+^ (39).

The stimulation of P2X7R leads to neurodegeneration through the release of several bioactive substances, including pro-inflammatory cytokines (IL16, IL1β, IL18, TNF-α) (46–48), chemokines (CCL3, CXCL2) (49, 50), proteases (Rac1, NADPH oxidase 2) (48), reactive oxygen species (ROS) (51, 52), and NO (53, 54), as well as excitotoxic glutamate (55, 56) or ATP (19). P2X7R acts as a crucial initiator of inflammation since microglia recognize pathogen-associated molecules (PAMP), such as lipopolysaccharide (LPS), or danger-associated molecular patterns (DAMP), like ATP (57). Upon exposure to these substances, P2X7R stimulates the release of the cytokine interleukin-1β (IL-1β) that facilitates the inflammatory response. Conversely, the inability of astrocytes to release IL-1β is attributed to NLRP3 deficiency (58).

## 3 P2X7-mediated signaling pathway

### 3.1 P2X7/MAPK

MAPKs, or mitogen-activated protein kinases, are serine/threonine kinases that regulate gene expression. This includes p38MAPK, ERK (extracellular signal-regulated kinase), and JNK (c-jun N-terminal kinase). They respond to extracellular stimuli and regulate various physiological processes, such as gene expression, mitosis, metabolism, cellular differentiation and motility, stress response, and cell survival or death (59, 60).

LPS can cause inflammation in microglia by activating the phosphorylation of three vital MAPK pathways - p38, JNK, and ERK - resulting in the secretion of pro-inflammatory cytokines (61). Researchers observed that inhibiting P2X7R antagonist brilliant blue G (BBG)and treating BV2 cells with LPS can halt MAPK activation by preventing the phosphorylation of p38MAPK, JNK, and ERK. This treatment decreased the secretion and expression of pro-inflammatory cytokines, such as IL-16, IL-1β, and TNF-α mRNA. Remarkably, using a MAPK inhibitor further intensified the inhibitory effect on MAPK. The findings indicate that BBG can effectively alleviate the neuroinflammatory response triggered by LPS in BV2 cells by inhibiting the MAPK signaling pathway (46). Inhibiting MAPKs led to significant neuroprotection in models of subarachnoid hemorrhage, cerebral hemorrhage, and PD (62, 63). ATP activates JNK, p38, and ERK. While JNK and ERK contribute to the production of TNF mRNA, p38 does not affect elevated TNF mRNA levels. Instead, it inhibits TNF mRNA transport from the nucleus to the cytoplasm and stimulates TNF release from microglial cells. The release is dependent on P2X7R, which may play a role in activating JNK and p38. Downstream from P2X7R, members of the tyrosine-protein kinase SRC (SRC) family (possibly PTK) are involved in activating JNK and p38 (15, 64). TNF release from microglia treated with 2’(3)-omicron-(4-Benzoylbenzoyl) adenosine-5’-triphosphate (BzATP) in neuron-microglia co-cultures reduced glutamate-induced neuronal cell death. P38 and JNK activation appears to be independent of inward Ca^2+^ flow (64), while a different study suggests a potential relationship between the inward flow of Ca^2+^ through P2X7R and the activation of p38 and JNK (65).

In a rat model of PD, the administration of LPS triggers an inflammatory response that results in the degeneration of dopaminergic neurons in the nigrostriatal pathway (66, 67). Microglial activation and the loss of dopaminergic neurons in the nigrostriatal system have been linked to enhanced expression of P2X7R in microglia and elevated levels of p38MAPK phosphorylation. The inhibition of P2X7R with BBG reduces microglial activation and prevents p38MAPK-induced degeneration of dopaminergic neurons (68). P2X7R antagonists effectively prevented the depletion of striatal dopamine stores induced by 6-hydroxydopamine (69, 70). Nonetheless, other studies found that P2X7R deficiency or inhibition did not impact dopaminergic neuron loss induced by 1-methyl-4-phenylpyridine or rotenone in chemical PD models (71). This lack of consistency might be ascribed to the extent of nigral damage induced by different models or the duration of P2X7R antagonist treatment.

Activation of P2X7 is associated with the AKT and ERK pathways, leading to cell death. However, it is not linked to the release of IL-1 family cytokines (IL-18, IL-1β, IL-1α) (47). In cortical astrocytes of rats, P2X7 activation induces AKT phosphorylation (72). However, the stimulation of P2X7 with BzATP in microglial cells results in ERK and AKT dephosphorylation (47), which might be attributed to their different cell types (73).

**Figure 3:**
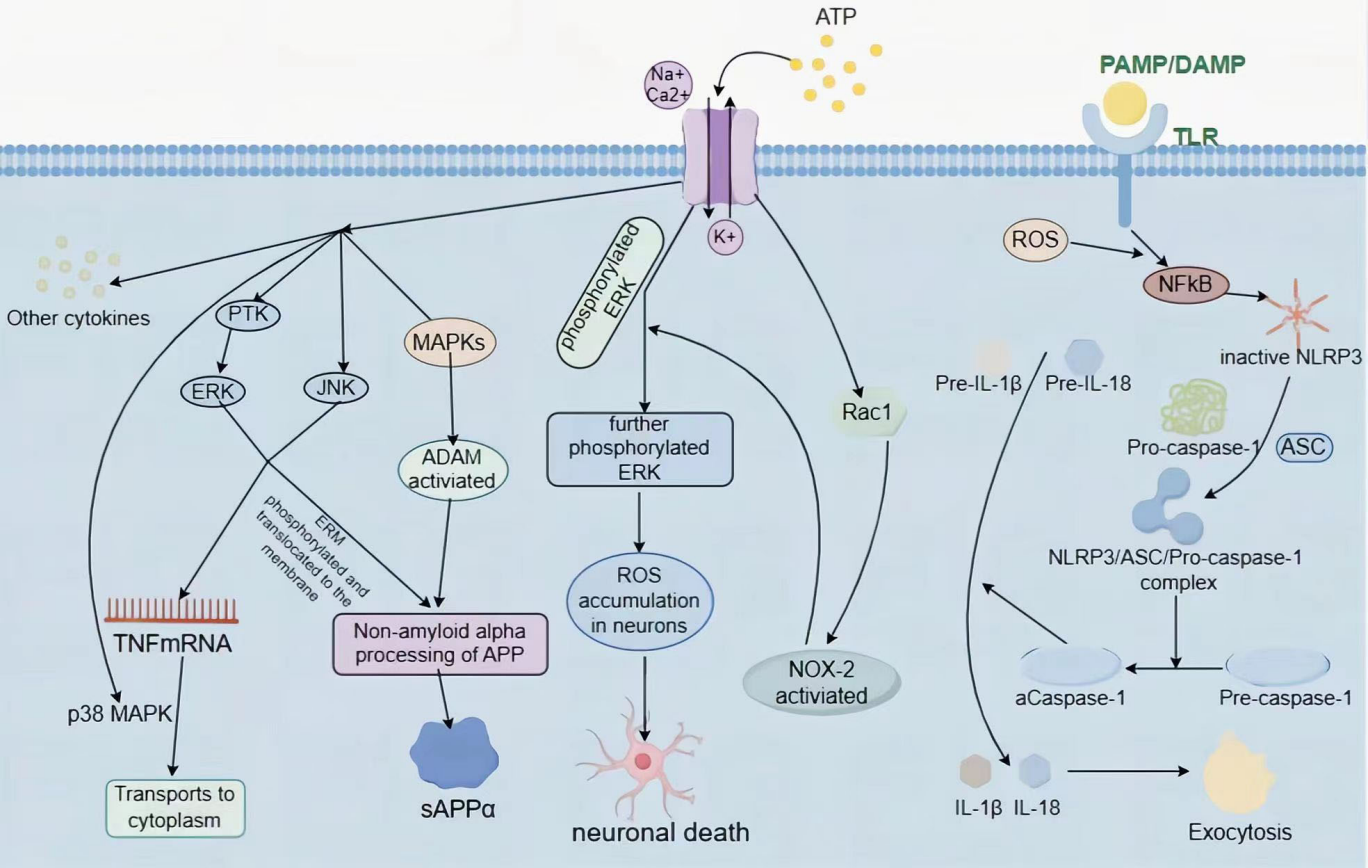
Overview of the P2X7-mediated signaling pathway. P2X7R triggers the activation of P38/ERK/JNK MAPK, leading to the build-up of TNF mRNA in the cytosol, which contributes to neuroinflammation. Moreover, sAPPα, released due to non-amyloid α cleavage of APP, is also impacted by P2X7R, following two distinct pathways, one ADAM-dependent and the other ADAM-independent. ROS can be toxic to neurons both inside and outside the cell, leading to neuronal death and promoting neurodegenerative disease progression. P2X7R mediates NOX-2 activation, resulting in the accumulation of ROS. P2X7R also stimulates the production of NLRP3 inflammatory vesicles and IL-1β secretion, resulting in pro-inflammatory effects outside of the cell.

Stimulating P2X7R activates MAP kinases, leading to increased a disintegrin and metalloproteases (ADAM) phosphorylation. These ADAMs, specifically ADAM9, -10, and -17, facilitate the non-amyloidogenic deformation α-processing of the amyloid precursor protein (APP) (74–76). Furthermore, a separate ADAM-independent process for APP α processing has been observed in mouse and human neuroblastoma cells, primary mouse astrocytes, and neural precursor cells. This process differs from the α-secretase activity of ADAM9, -10, and -17 in response to APP, primarily leading to α-cleavage. Moreover, it promotes the release of the soluble ectodomain of APP (sAPPα) while inhibiting the production of sAPPβ and amyloid β (A-β) peptides. This process involves the Erk1/2 and JNK pathways and is dependent on P2X7R (77). Another study on glioma U251 cells also reported the joint involvement of Erk1/2 and JNK in APP α-cleavage (78). Furthermore, stimulation of P2X7R leads to ERM (Ezrin/Radixin/Moesin) phosphorylation, which relocates to the plasma membrane and interacts with P2X7R. This interaction causes APP processing and subsequent protein hydrolysis, resulting in sAPPα shedding dependent on P2X7R. Additionally, P2X7R signaling triggers ERM phosphorylation in Neuro2a cells via upstream Rho kinase and MAPK activation, while downstream PI3K activity is stimulated (79) (**Figure 3**).

### 3.2 P2X7/ROS

Mitochondrial damage from inflammation and metabolic stress not only impairs energy production but also triggers the accumulation of ROS. This ultimately causes neuronal cell death and exacerbates the progression of neurodegenerative conditions (80, 81). The primary cause of progressive neuronal death is thought to be the inflammatory response that results from the activation of microglia, which causes ROS to accumulate (82, 83). ATP-induced neurodegeneration and oxidative stress are significant contributors to neurodegenerative diseases due to P2X7R-mediated mitochondrial dysfunction and inward Ca^2+^ flow into neurons (84–86).

Mitochondrial dysfunction results in decreased ATP production, Ca^2+^ dysregulation, and the generation of ROS. Mitochondria produce superoxide, a significant source of reactive oxygen species during ischemic and hypoxic conditions at the respiratory chain’s origin (85). Reactive oxygen species can damage macromolecules in the plasma membrane of neurons through oxidative modifications and harm (87). Activation of P2X7R by α-synuclein causes a reduction in mitochondrial membrane potential and an increase in the production of mitochondrial ROS (84). Subsequently, ROS stimulate the mitochondria-dependent intrinsic apoptotic pathway and activate pro-apoptotic proteins (88), which cause mitochondrial dysfunction, decreased cellular energy production, and cell death (89).

Oxidative stress is mainly characterized by increased levels of ROS and reduced antioxidant system capacity to combat free radicals (90–92). Oxidative stress can modify the inflammatory response in multiple ways, activating transient receptor potential (TRP) channels, specifically TRPV1, and signaling pathways (93). Additionally, stress can activate other mechanisms that increase the secretion of pro-inflammatory mediators (94), resulting in neuronal damage (95). Both oxidative stress and mitochondria significantly impact triggering apoptosis, where mitochondria serve as a source and target of ROS (96).

NADPH oxidase 2 (NOX2 or phagocytic oxidase PHOX) is a significant contributor of extracellular and intracellular ROS in microglia (97). The generation of ROS is a natural byproduct of cellular metabolism and plays a role in intracellular and extracellular signaling (98). Extracellular ROS are harmful to neurons, while intracellular ROS function as signaling mechanisms in microglia, activating p38 and ERK1/2 to prompt the production of various pro-inflammatory and neurotoxic cytokines, such as tumor necrosis factor-α, prostaglandin E2, and IL-1β (99). In SOD1-G93A mice, the absence of NOX2 enhances disease progression and survival (48).

In SOD1-G93A microglia, BzATP stimulates P2X7R, activating Rac1 (48, 98). Rac1 is a crucial activator of NOX1 and NOX2 from the Rho GTPase family (100). The activation of Rac1 enhances NOX2 activity, leading to an increase in ROS generation (48). P2X7R-mediated activation of NOX2 and consecutive ROS generation in microglia relies entirely on Rac1 (48). Additionally, phosphorylation of ERK1/2 increased in ALS microglia triggered by ATP stimulation of P2X7R. There exists an interdependence between the NOX2 and ERK1/2 pathways that combine to produce ROS. NOX2 activation results in further ERK1/2 phosphorylation, causing excessive ROS production (48, 101). Intrathecal injection of BzATP results in spinal ROS production and oxidative DNA damage in dorsal horn neurons (98).

Activation of P2X7R induced ROS production and IL-6 release in spinal cord astrocytes. Both releases partially passed through NADPH oxidase. The P2X7R antagonist A438079 inhibited ROS increase, whereas the P2X7R scavenger N-acetylcysteine (NAC) partially inhibited BzATP-mediated IL-6 release (52). Meanwhile, P2X7 activation induced ROS formation in EOC13 cells, leading to subsequent cell death (102, 103). ROS formation occurs through a mechanism independent of Ca^2+^ inward flow and K^+^ efflux (104). In contrast, P2X7-induced ROS formation in primary rat microglia depends on Ca^2+^ inward flow (105).

ROS-induced oxidative stress is a significant factor in the development of AD (106). ATP released from microglia, stimulated by A-β, activates NADPH oxidase-mediated ROS production through P2X7R (105). The upregulation of P2X7R activation and ROS production coincides with A-β growth, and increased levels of oxidative stress are strongly linked with synaptic loss in the AD mouse model mediated by A-β (51). This potentially explains the microglia-induced neuronal damage in the brain affected by AD.

### 3.3 P2X7/NLRP3 inflammasome

The production of pro-inflammatory cytokines has been associated with several neurodegenerative diseases, such as AD, PD, and MS (107). In animal models, the evidence suggests that inflammation may contribute to disease progression but not necessarily be the primary initiator of neurodegenerative diseases (108). The NLRP3 inflammasome comprises the sensory protein NLRP3, the junction protein (ASC), and effector proteins (caspase-1) (109). The activation of the NLRP3 inflammasome occurs through a two-step process consisting of “initiation” and “activation” (110). The process that ignites the activation of inflammatory vesicles is called “initiation.” Initiation is instigated by Toll-like receptors (TLRs) that recognize PAMPs, DAMPs, environmental stress, or by NF-κB which is activated by TNF-α (111). Moreover, ROS are indispensable for NF-κB activation (112). Following this, NF-κB induces the upregulation of expression levels of NLRP3, pro-IL-1β, and pro-IL-18 (113). However, NLRP3 remains inactive (114). The second signal is called “triggering” or “activation”. NLRP3 and ASC form a complex with Pro-caspase-1 under certain conditions, leading to the conversion pro-caspase-1 to caspase-1. The activated caspase-1 converts pro-IL-1β and pro-IL-18 to their active forms, which are then released extracellularly to promote pro-inflammatory effects (115). IL-1β is produced in response to stimulation from various inflammatory vesicle activators, such as ATP, Nigericin, and alum. Furthermore, microglia release IL-18 and IL-1α. However, functional NLRP3 inflammatory vesicle formation and IL-1β secretion are unique to microglia in the mouse brain and do not occur in astrocytes (58).

## 4 P2X7R and neurodegenerative diseases

With an aging population, the worldwide prevalence and rates of disability associated with neurodegenerative diseases are increasing, significantly impacting societal development and progress. This section provides a detailed analysis of the major neurodegenerative diseases, including AD, PD, HD, MS, and ALS. Neuronal damage is the main pathological feature of the above-mentioned neurodegenerative diseases (116). While the five disorders have diverse origins (**Table 1**), they share a common characteristic: chronic inflammatory damage in the CNS, which results in the persistent activation of innate immune cells. This includes the infiltration of peripheral immune cells across the blood-brain barrier (BBB), which is observed in MS (116). Moreover, there is no doubt that P2X7R, which is present in high densities in microglia (12), astrocytes (134), and oligodendrocytes (135), largely determines changes in neuronal function and activity in the CNS. Furthermore, in addition to amplifying inflammatory damage through glial cells (19), P2X7R receptors on the surface of neurons themselves can actively induce autophagy in neurons (136).

**Table 1:** Overview of common neurodegenerative diseases.

Disease	Pathogeny	Immune response	Neuropathological features	Clinical manifestation	Incidence (number/100 000)	Forecast growth rate	References
AD	AD – misfolded and aggregated tau and APP	↑TNF‐*α*, ↑IFN‐*γ*, ↑chemokines, ↑complement, ↑TLRs, ↑antibody , ↑T‐cells , ↑activated microglia	Extracellular amyloid plaques, intracellular neurofibrillary tangles and nerve cell death	Language, visual space and executive dysfunction	9330	↑3.3% per year (triple by 2050)	(117–121)
PD	Selective loss of dopaminergic neurons in substantia nigra due to *α*‐syn‐ intraneuronal inclusions	↑TLRs, ↑CD14, ↑IL‐1*β*, ↑IL‐6, ↑TNF‐*α,*↑T‐cells, ↑antibody , ↑activated NK cells,↑ activated microglial	neural inclusions in the form of Lewy bodies and Lewy neurites with cell loss in the substantia nigra and other brain areas	Bradykinesia,rigidity,tremor,gait alterations	100–200	Double in 25 years	(119, 122–124)
HD	Autosomal dominant genetic disease,expansion of CAG (Q) in huntingtin gene induces aberrant toxic protein	↑microglial proliferation, ↑complement	General atrophy of the brain and degeneration of the striatum (caudate nucleus and putamen), accompanied by specific loss of efferent spinous neurons (MSN)	Motor defects (chorea , loss of coordination), Mental symptoms(depression, mental illness and obsessive-compulsive disorder)	0.02–9.71	↑15–20% per decade	(119, 125, 126)
MS	Autoimmune viral	↑ROS,↑HSPs, ↑neurotrophins, ↑complement, ↑innate receptors,↑cytokines, ↑chemokines, ↑activated microglial , ↑activated macrophage, ↑antibodies , ↑T‐cells	Demyelination and axonal degeneration	Optic neuritis (optic nerve inflammation), Uhthoff phenomenon (MS symptoms fluctuate or worsen briefly as body temperature increases) and Lhermitte phenomenon (abnormal shock-like sensation of the spine or limbs during cervical flexion)	9.64	↑2.4% per year	(119, 127–129)
ALS	Aberrant aggregated proteins due to mutations SOD1, TDP; C9orf72 or FUS genes	↑Complement, ↑CD14, ↑macrophages, ↑IL‐6, ↑TNF‐*α*	Extensive loss of lower motor neurons in the anterior horn of the spinal cord and brainstem, degeneration and loss of Betz cells (macropyramidal neurons) in the primary motor cortex, degeneration of the lateral corticospinal tract, and reactive gliosis	Leg and arm distal progressive unilateral weakness, no remission or recurrence. Atypical manifestations include emotional instability, frontal lobe cognitive impairment, weight loss, muscle bundle tremor and painful spasm, and no muscle weakness.	1.9	↑69% in 25 years	(119, 130–133)

“↑” implies an upward adjustment.

### 4.1 P2X7R and Alzheimer’s disease

AD is a neurodegenerative disorder characterized by neuronal fibrillary tangles and senile plaques. Neuronal fibrillary tangles result from accumulations of hyperphosphorylated tau protein inside neurons, while extracellular A-β peptides lead to the formation of senile plaques (137). Nevertheless, the degree of cognitive impairment in AD does not correlate with the presence of amyloid plaques or neuroprotective fibril tangles (138). Most animal disease models are transgenic mice resulting from random mutations in genes that encode proteins associated with AD pathology or rodents injected with A-β into their brains (139).

P2X7R is involved in various processes, such as APP processing to produce A-β (140), synaptic dysfunction, oxidative stress (51), and neural inflammation (105). Specifically, in the production of A-β, P2X7R may be involved in the cleavage of APP, which has three different secretases - α-, β-, and γ-, cleaving at different sites on APP. The β segment produces A-β and γ-secretases present in amyloid plaques in AD patients’ brains. When α-secretase processes APP in a non-amyloidogenic manner, it leads to hydrolysis of A-β peptide sequences and shedding of sAPPα, which has neuroprotective and neurotrophic effects (141).

Stimulation of P2X7R activates ADAM9, -10, and -17, which have α-secretase activity (74, 75). They mediate the non-amyloidogenic processing of APP (76). Additionally, P2X7R triggers a new non-amyloidogenic pathway independent of ADAM9, -10, and -17 (77), promoting a significant shift in APP processing towards α-cleavage. This process increases the release of sAPPα while inhibiting the production of sAPPβ and A-β peptides (77). Stimulation of P2X7R-induced release of sAPPα was observed in human APP-expressing mouse and human neuroblastoma cells, mouse primary astrocytes, and neural progenitor cells. The knockdown of P2X7R could inhibit this release through a P2X7R antagonist or specific small interfering RNAs (siRNAs). It was not observed in neuronal cells from P2X7R-deficient mice (77). Inhibitors of β- and γ-secretase have been extensively researched, but therapy using these inhibitors is limited by associated side effects resulting from reduced β-secretase 1 and γ-secretase activity. Therefore, alternative approaches to treating AD, such as modulation of α-secretase activity, are being explored (142).

When extracellular particles, specifically A-β peptides (143), are present, ATP is released from damaged neurons, microglia, and astrocytes (144). High levels of extracellular ATP activate P2X7R, which is expressed at significantly higher levels during microgliocytosis and significant cognitive and motor impairment (145). A similar phenomenon was observed in microglia surrounding A-β in patients with Alzheimer’s disease (AD), and AD mouse models (146), and the increase in P2X7R levels mirrored the progression of AD (147). Additionally, activating P2X7R boosted microglia migration towards senile plaques while simultaneously inhibiting phagocytosis (147). In familial Alzheimer’s disease (FAD), the aggregation of A-β peptide occurred prior to P2X7R expression in microglia. This was found by investigating a new transgenic mouse model - P2X7R-EGFP/J20 mice. Furthermore, microglia expressing P2X7R were closer to emerging plaques than those without P2X7R expression133.

P2X7R produces chemokines induced by A-β peptides and recruits CD8T cells in brain parenchyma. Chemokines are expressed excessively *in vitro* and AD mouse models in response to A-β peptides, leading to the inflammatory process and recruitment of immune cells (148). Furthermore, this overexpression of chemokines contributes to the subsequent neurodegenerative process (149).

In the Neuro-2a cell line expressing APP, activation of the P2X7R receptor results in decreased α-secretase activity, whereas activation of the P2Y2 receptor leads to the opposite effect. In cultured cerebellar granule neurons, activating P2X7R inhibits and neuroprotects glycogen synthase kinase 3 (GSK-3), the less active form of α-secretase (150). In J20 mice, a transgenic model of FAD that expresses the human mutant APP protein, inhibiting P2X7R *in vivo*, reduced the number and size of hippocampal A-β significantly. This reduction was facilitated by an increase in the phosphorylated form of GSK-3, which, in turn, enhanced the α-secretase-induced non-amyloidogenic degradation of APP (150). Multiple lines of evidence suggest that the inhibition of α-secretase by BzATP is dependent solely on P2X7R. To prevent the inhibitory effects of BzATP on α-secretase activity, both the pharmacological blockade and the synthesis inhibition of P2X7R with RNA interference be effective (142). Several studies demonstrate that a lack of P2X7R restores hippocampal synaptic integrity and plasticity, rescues memory deficits in APPPS1 mice, and reduces A-β pathology. However, the effects are not modulated by the sAPPα pathway, IL-1β treatment, microglial activation, or phagocytosis (12).

### 4.2 P2X7 and Parkinson’s disease

PD is the second most common neurodegenerative disorder globally, affecting over 6 million individuals (151). It is also a leading contributor to neurological disabilities. Typical symptoms of PD consist of bradykinesia, resting tremor, tonus, and changes in posture and gait, significantly impacting patients’ quality of life (95). The pathological characteristics of PD involve the creation of neural inclusion bodies that comprise eosinophilic material, Lewy vesicles, and the demise of dopaminergic neurons with injury to the central region of nigrostriatum (122). Misfolded α-synuclein aggregates predominantly constitute the Lewy bodies among these characteristics (122).

After an extensive investigation, it was found that postmortem PD patients have an elevated number of reactive microglia with phagocytic activity in their brains (152). Furthermore, similar results as well as high expression of P2X7R were observed in a mouse model of PD (153), emphasizing the strong correlation between microglia-induced neuroinflammation and PD in this disease. P2X7R plays a significant role in the pathogenesis of PD, as it produces a profoundly pronounced facilitatory effect. When neuroinflammation occurs, dying neurons release a significant amount of ATP, which then activates the P2X7R located on the surface of glial cells. The P2X7R induces a positive feedback loop of Ca^2+^ influx, promoting the opening of P2X7R and pannexin-1 channels on the membrane surface, which then releases more ATP (154). This increased ATP release also boosts the exocytosis of K^+^ ions and triggers the assembly of NLRP3 inflammasomes. Subsequently, the activation of caspase-1 cleaves pro-IL-1β to IL-1β, thus facilitating its release (155). As a result, neuroinflammation worsens, and this ultimately accelerates neuronal death.

Several studies have identified the interaction between the pathological protein α-Syn and microglia as a crucial factor in the neuroinflammatory process of PD. Alpha-synuclein not only binds to microglial NOX2, thereby activating the NOX complex and triggering oxidative stress *in vivo* (156) but also directly activates P2X7R on its surface to exert its effects; this activation persists in the presence of exogenous ATP withdrawal (157). During this process, activated microglia release excitotoxic glutamate, which damages dopaminergic neurons through ATP/glutamate secretion on the one hand (158), and destroys dopaminergic neurons through the release of ROS on the other hand, leading to the extensive destruction of dopaminergic neurons and degeneration, thus triggering the development of PD (159). In addition, the pathological changes in PD are closely related to excessive Ca^2+^ inward flow due to P2X7R activation, and the high intracytoplasmic calcium environment also directly induces apoptotic necrotic loss of dopaminergic neurons (160). Moreover, other studies have demonstrated that Ca^2+^ binds to the C-terminus of α-Syn to increase its localization in presynaptic terminals and synaptic vesicles (161).

A study demonstrated that BBG, a P2X7R antagonist, successfully reduced neuronal apoptosis in rat subarachnoid hemorrhage by inhibiting p38MAPK (162). Peroxisome proliferator-activated receptor-γ (PPARγ) coactivator 1α (PGC-1α) is a protein that interacts with nuclear receptors like PPARγ, which negatively regulates the transcription of the NF-KB pathway, a pro-inflammatory pathway, thereby inhibiting inflammatory responses. In contrast, PGC-1α regulation is accomplished through phosphorylation at multiple sites by several phosphokinases, such as MAPK and AKT (163). Another experiment simulating PD treatment observed that attenuation of DA neuronal damage in an LPS rat model of PD was achieved by inhibiting the P38MAPK pathway with a P2X7R antagonist. The criterion for efficacy was counted using tyrosine hydroxylase-immunoreactive (TH-ir) neurons in the substantia nigra. The study revealed a significant reduction in TH-ir levels in the LPS-treated group. After 15 days of treatment with BBG, the LPS group witnessed an effective reversal of the reduction in TH-ir levels (68). The studies above highlight the critical role played by P2X7R-mediated stimulation of oxidative stress via the MAPK pathway in promoting neuroinflammation during the pathogenesis of PD. In PD animal model experiments, the nigrostriatal region of rats was damaged with 6-OHDA to induce PD. Apomorphine caused the rats to exhibit rotating behavior, confirming the establishment of the animal model of PD damage. In batches, the rats were treated with BBG. The number of rotations per minute of the saline-treated rats remained unchanged from seven days prior. However, the BBG-treated rats significantly decreased the number of rotations, providing further evidence that BBG treatment alleviates motor deficits in lateralized parkinsonism (163). Additional studies suggest that the P2X7R-mediated neuronal cell swelling and necrosis may be linked to abnormal functioning of the substantia nigra striatal region in PD. When the SN4741 neuronal cells derived from transgenic mouse embryos were exposed to high concentrations of ATP, their cell volume increased dramatically and in a concentration-dependent manner within twenty minutes. Subsequently, the cells exhibited necrotic manifestations typical of cellular necrosis, such as nuclear swelling, DNA leakage, ER integrity loss, and cytoplasmic vacuole formation (164). Furthermore, prior research has indicated that the sensitivity of P2X7R to ATP activation amplifies with decreasing levels of extracellular divalent cations. This implies that a positive feedback loop occurs when there is neuroinflammation in the substantia nigra striatal region, as lower concentrations of ATP activate P2X7R, worsening the lesion. In summary, ATP binding to the P2X7R stimulates microglia and recruits peripheral immune cells through a variety of complex pathways. These pathways include induction of calcium influx, activation of NLRP3 inflammatory vesicles that induce cell death and tissue damage, and release of more ATP (19). The ATP release/P2X7R activation/apoptosis axis then acts in a positive feedback loop, which contributes significantly and continuously to the neurodegenerative process of PD (19).

### 4.3 P2X7 and Huntington’s disease

HD is an autosomal dominant neurodegenerative disorder characterized by motor, cognitive, and psychiatric deficits (165). Commonly observed motor symptoms include chorea and coordination difficulties (166). The primary cause of HD is believed to be the amplification of a CAG repeat in the first exon of the huntingtin gene (HTT), leading to the production of the mutant Huntington’s protein (mHTT) (167). The expansion of a bundle of polyQ in the N-terminal segment of the encoded protein, due to CAG repetition, causes abnormal folding of mHTT and its accumulation in brain cells (168). Subsequently, earlier transcriptional dysregulation occurs, along with abnormalities in synaptic and axonal transport, disruption of the protein homeostasis network, aggregation pathology, compromised function of the nuclear pore complex, oxidative damage, mitochondrial malfunction, and extrasynaptic excitotoxicity (169, 170).

There is now extensive direct evidence suggesting that pathways mediated by the P2X7R contribute to the development of Huntington’s chorea. This makes it a potentially interesting target for HD patient treatment. It is worth noting that the increase in the number of CAG triplet repeats is not related to either the transcriptional process of P2X7 gene expression or the role of the P2X7R (171). However, research has suggested that the expression of HTT mutant genes may render neurons more vulnerable to P2X7R-mediated apoptosis, effectively increasing susceptibility (171). In this experiment, it was observed that Tet/HD94 mutant mice showed a significant decrease in the survival of their cortical and striatal neurons when exposed to 10 Mm BzATP, whereas this ATP analogue had no discernible effect on wild-type mice (171). Administration of BBG, a P2X7R receptor antagonist, was discovered to slow or prevent the negative impacts of ATP analogues on neuronal viability in mice with the HTT mutant phenotype (171). The administration of BBG to R6/1 HD gene mouse models also had a similar effect, improving motor parameters and alleviating weight loss after treatment (171).

In a certain centralized investigative study of HD patients, it was shown that there was a significant fold increase in P2X7R protein levels (including protein bands of all four isoforms of P2X7R-A, B, H, and J) in affected individuals (172). In addition, immunohistochemistry revealed that more diffuse and intense reactivity and a greater number of immunoreactive cells can be observed in striatal sections of HD patients (172).

A reported study has demonstrated that after using BzATP in extracellular electrophysiology experiments in cortical striatal slices from both wild-type mice (WT) and R6/2 (HD-type mice), a decrease in FP amplitude was induced (173). Nonetheless, BzATP’s reduction in FP proved much more statistically substantial in transgenic mice than in WT mice (173). This indicates that the ATP-activated P2X7R pathway hinders synaptic transmission to a greater extent in HD genotypes. This could contribute to the gradual impairment of neuronal viability in HD patients. Moreover, there is evidence of mHTT mutant protein accumulation in astrocytes of both HD patients and animal models. This accumulation is linked to reduced astroglial potassium channel (Kir4.1) (174). In the R6/2 mouse model of HD, it has been demonstrated that astrocytes exhibit anomalous electrophysiology and significantly elevated extracellular levels of potassium ions (174). These manifestations may stem from irregular ion exchange caused by the opening of large pores influenced by the activation of P2X7R (174).

Regardless, the activation of P2X7R is highly significant in initiating an expedited process of neuronal degeneration within the striatal region during the onset and advancement of the disease in Huntington’s patients. This could be a pivotal breakthrough in treating individuals with HD.

### 4.4 P2X7 and multiple sclerosis

MS is a chronic inflammatory disease of the CNS that is mediated by autoreactive helper T cells (Th1 and Th17 cells). Patients with MS are typically between the ages of 20 and 40, and it is the leading cause of disability among young people in the United States and Europe (175). MS is characterized by demyelination and axonal degeneration (176). Neurological symptoms may happen during seizures, including weakness, altered sensation, balance disturbances, visual impairment, and color vision or diplopia (127). The critical pathological characteristic of MS is the emergence of inflammatory plaques, causing damage to the myelin sheaths and specialized cells (e.g., oligodendrocytes) in both the white and gray matter of the brain and spinal cord, leading to neuronal loss. Upon initial exposure to unfamiliar antigens, Th1 cells produce pro-inflammatory cytokines IL-1 and IFN-γ, while Th17 cells produce IL-17 (127).

The pathology of MS is primarily driven by the interaction of neuroglial cells and autoreactive immune cells. Activation of P2X7R on astrocytes and microglia, which mediates purinergic signaling, has been suggested as a significant causative factor in these pathologic processes (4). Some experimental studies have indicated a significant presence of P2X7-immunoreactive microglia/macrophages in the spinal cord of patients with MS, particularly in the dorsolateral white matter region of the degenerating corticospinal tracts (177). Additionally, inflammation-related substances such as IL-β and COX-2 are also heightened in the affected areas (177).

Cell death is thought to raise extracellular ATP levels (178), boosting P2X7R expression in microglia, which sequentially releases IL-1β, COX-2, and PGE2, ultimately leading to additional cell death and ATP release. This cascading cyclic mechanism could play a significant role in the demyelination process observed in MS patients (177). In a study conducted on SP(Secondary progressive)-type MS patients, researchers found that astrocytes in the frontal cortical region had an increased expression of P2X7R and promoted neuroinflammation in a manner similar to microglial cells. Additionally, they discovered that direct activation of P2X7R by BzATP increased MCP-1 (Monocyte chemoattractant protein-1) levels in astrocytes, a protein that is responsible for leukocyte recruitment during MS progression (179). In astrocytes, P2X7 activation induces phosphorylation of ERK1/2 and activation of the PI3/AKT signaling pathway, both of which contribute to promoting neuroinflammatory responses (180). Moreover, exposure to high concentrations of ATP or the selective agonist BzATP acting on P2X7R stimulates the shedding of MPs (microparticles) from particulate vesicles on the surface of microglia or astrocytes. Exocytosis of MP results in the release of significant quantities of pro-inflammatory factors, including IL-1β, which expedites the neuroinflammatory process in MS development (181). As previously stated, MS is a demyelinating disease that centers on the death of oligodendrocytes. According to a study, ATP accumulation triggers Ca^2+^ signaling in oligodendrocyte progenitor cells (OPCs), leading to the activation of the P2X7R and P2Y1R pathways. This impacts the cells’ growth, development, differentiation, and other processes, potentially acting as a mechanism for the onset of demyelination (182).

There are numerous immune cell types that participate in the development of pathogenic neuroinflammation in MS. Among them, the monocyte macrophage lineage expresses the P2X7R at the highest level. Activated monocytes are often one of the first phenotypes to arrive at the site of CNS neuropathy (183). In a particular study on patients with MS, it was discovered that despite a significant increase in P2X7R mRNA in total cell extracts from the frontal cortex in SPMS (Secondary progressive multiple sclerosis) patients, there was an unexpected decrease in P2X7R expression on the surface of monocytes (184). It is hypothesized that monocytes decrease P2X7R protein expression when the efficiency of excess toxic ATP removal reduces, efficiently preventing diminished viability caused by calcium overload. This sustains their activity for better participation in neuroinflammation (184).

Although definitive studies have not confirmed the inevitability of MS occurrence with the presence of P2X7R, many experiments have shown that deleting P2X7R can significantly reduce the incidence of EAE disease in mice. One study discovered that P2X7-deficient mice had significantly lower mean scores of clinical symptoms in comparison to WT mice, notwithstanding the insignificant alteration of the mean number of days of disease onset. This finding effectively implies that the deletion of P2X7R reduces the incidence of EAE (encephalomyelitis) (184).High levels of astrocyte activation were detected in various areas of white matter in WT EAE mice as well as in Bergman’s radial glial fibers. However, the level of activation was not significant in the P2X7null group (184).

Furthermore, related studies have identified a twofold function of P2X7R in the development of MS. The studies have demonstrated that the activation of P2X7R on erythrocytes can disrupt the regulation of cation fluxes, which are responsible for maintaining extracellular K-ion homeostasis and removing excessive toxic glutamate. These findings contribute to the intricate nature of the relationship between P2X7R and MS (185).

### 4.5 P2X7 and amyotrophic lateral sclerosis

ALS is a long-term neurological disorder defined by the invasion of lesions, mainly in the anterior horn cells of the spinal cord, brainstem motor nerve nuclei, and the pyramidal tract. This can lead to the loss of upper and lower motor neurons in the motor cortex, brainstem nuclei, and anterior horn of the spinal cord (186). This condition primarily affects the motor system and presents accompanying symptoms, such as skeletal muscle atrophy, progressive paralysis, respiratory failure, and death within 2-5 years (187). The pathophysiology of ALS is characterized by neuromuscular junction loss in both the upper and lower motor neurons, axonal retraction, subsequent cell death, astrocytic hyperplasia, and an increase in microglia around the lesion (188).

Neuroinflammation is a key pathological mechanism in ALS, resulting from the activation of P2X7R and leading to chronic microglial activation (189). This is considered a mechanism that contributes to the death of motor neurons (190). In addition, impaired autophagy can also lead to the damage and death of motor neurons (191). Activation of P2X7 *in vitro* exacerbates pro-inflammatory responses, including NOX2 activation in microglia, elevated levels of TNF-α, COX-2, MAPK, and neuronal toxicity (48). Stimulating P2X7R using the P2X7R-specific agonist BzATP before the onset of pathological neuromuscular symptoms in SOD1-G93A mice resulted in enhanced muscle fiber innervation and metabolism, preserved neuromuscular junction morphology, and stimulated satellite cell proliferation and differentiation. This intervention effectively prevented skeletal muscle denervation in SOD1-G93A mice (192). During the presymptomatic stage of ALS disease, administering BzATP via intramuscular injection improved locomotor activity in mice by revitalizing muscle cells and infiltrating macrophages. The treatment not only protected the retrograde propagation of skeletal muscle to the CNS but also enabled direct and immune-mediated protection. Additionally, it reduced neuroinflammation and promoted spinal motor neuron survival (193).

Repeated stimulation of spinal astrocyte P2X7R with ATP or BzATP activates it, leading to a neurotoxic phenotype that causes motor neuron death. Conversely, inhibiting P2X7R or apyrase, an enzyme that degrades ATP, by using BBG eliminates their toxicity to motor neurons (194). Brief stimulation of the P2X7R initiated autophagy activation and enhanced the expression of anti-inflammatory biomarkers in microglia of SOD1-G93A mice (M2 microglia). In contrast, prolonged activation of P2X7R caused disruption of autophagic fluxes, which could lead to a shift towards a pro-inflammatory phenotype (M1 microglia). These results indicate a dual function of the receptor in the pathway (195).

Previous research suggests that P2X7R ablation accelerates clinical onset and worsens disease progression in mSOD1 mice (196). In contrast, BBG-induced P2X7R antagonism suppresses microglial proliferation, alters microglial-associated inflammatory genes, enhances motoneuron survival, mildly delays onset, and improves motor function but does not impact survival (197).

In the SOD1-G93A mouse model, BBG was used to antagonize P2X7R, resulting in reduced neuroinflammation and promotion of the survival of lumbar medullary motor neurons. This may ultimately delay the onset of ALS in a mouse model of ALS (198). Similarly, AXX71 and AXX13 were found to reduce proinflammatory markers, delay the onset of neuromuscular injury, and transiently preserve motor function and muscle strength by antagonizing P2X7R (199). The results indicate that P2X7R may play a role in ALS, a neurodegenerative disease. P2X7R antagonists have the potential to delay the onset and reduce the clinical symptoms of ALS.

### 4.6 Other diseases

In addition to the five neurodegenerative diseases mentioned above, P2X7R may also play a role in neuropsychiatric disorders, including depression, anxiety disorders, and post-traumatic stress disorder (PTSD). These disorders are a frequent cause of death in the elderly and are characterized by behavioral changes (200).

Depression is a prevalent psychiatric disorder characterized by persistent sadness, anhedonia, and diminished interest, which can lead to impairment of daily functioning (201). The onset or development of depression may be influenced by neuroinflammation (202). The involvement of the pro-inflammatory cytokine IL-1β, released by microglia, induces the secretion of corticotropin-releasing hormone (CRH), which in turn secretes adrenocorticotropic hormone (ACTH) and cortisol, along with a large number of other cytokines/chemokines, leading to mood dysregulation. Therefore, microglia may be a potential target for the treatment of depressive symptoms. Hyperactivation of P2X7 leads to increased release of inflammatory cytokines, such as IL-1β, which are involved in depression. Inhibiting P2X7R expression in the hippocampus, spinal cord, and dorsal root ganglia may alleviate visceral pain and depression. In a rat model of bone cancer, cancer-complicated pain and depression-like behavior were reduced by intrahippocampal injection of the P2X7R antagonist A438079. Microinjection of the P2X7R antagonist A-438079 into the amygdala significantly attenuated depressive and anxiety-like behaviors in neuropathic pain. This effect may be attributed to the antagonist’s inhibitory effects on microglia and astrocytes. These findings suggest that P2X7R may play a role in depression complicated by various kinds of pain. Additionally, salidroside, a bioactive extract from Rhodiola rosea L, may mediate depression by inhibiting P2X7/NF-KB/NLRP3-mediated focal death.

Anxiety is defined as excessive fear, anxiety, or avoidance of perceived threats. Studies have shown that ATP/P2X7R-initiated microglia in the ipsilateral hippocampus can drive anxiety-depressive-like behaviors associated with trigeminal neuralgia via IL-1β. P2X7 is also involved in brain monocyte aggregation associated with repeated social failure, IL-1β mRNA expression in enriched myeloid cells, plasma IL-6, and anxiety-like behavior. Additionally, IL-1β accumulation plays a role in the pathophysiology of PTSD. However, the relationship between P2X7R and PTSD has not yet been definitively investigated.

## 5 Potential therapeutic targets for neurodegenerative diseases: P2X7R antagonists

P2X7R are a major therapeutic target in the treatment of neurodegenerative diseases. To categorize common P2X7 antagonists, we have identified five groups (**Table 2**). The first group comprises divalent cations like Ca^2+^, Mg^2+^, Zn^2+^, and Cu^2+^. These cations hinder the activation of ATP-induced P2X7R. Experiments have demonstrated that their *in vitro* values of IC50 (μM) were 2900, 500, 11, and 0.5 at pH 6.1. A decrease in pH modifies the charge on histidine residues and prevents ATP-gated currents. In AD and PD, Mg^2+^ administration may be a potential strategy to reduce the deleterious effects of Ca^2+^ induced neuroinflammation.The second group, consisting of nonselective P2XR antagonists such as sulforaphane, RB-2(Reactive Blue 2), PPADS (pyridoxal phosphate-6-azobenzene-2′,4′-disulfonic acid), and iso-PPADS, exhibit lower potency and selectivity for the P2X7R as compared to other P2Rs. Furthermore, some other P2R antagonists display moderate selectivity for P2X7R while also having either low potency, as seen in oxATP(oxidized ATP), or high potency, as seen in BBG. OxATP inhibits P2X7 activation in microglia from both AD and non-demented brains, indicating therapeutic potential for oxATP in AD (146). BBG blocks rP2X7 at 10 nM concentration, and human P2X7R (hP7X2R) at 200 nM concentration (203, 204). Additionally, BBG blocks voltage-gated sodium channels at low micromolar concentrations (205). In the J20 hAPP transgenic mouse model of AD, BBG inhibits glycogen synthase kinase 3-β (GSK-3β) via P2X7R. This increases α-secretase activity in hippocampal neurons, thereby reducing amyloid-beta (Aβ) and subsequent plaque production (206). In an animal model of AD, BBG reduced levels of purinergic receptors, decreased gliosis, and mitigated blood-brain barrier leakage. Additionally, it exhibited neuroprotective properties and acted as an antagonist to the inflammatory response triggered by the P2X7R agonist 2’,3’-(benzoyl-4-benzoyl)-ATP (207). *In vivo* data obtained from the administration of BBG in HD mice strongly suggests that BBG prevents neuronal apoptosis and attenuates weight loss and motor coordination deficits. Alterations in P2X7 receptor levels and function are believed to contribute to the pathogenesis of HD.A third category of organic cations, including calmidazolium and 1-[N,O-Bis(5-isoquinolinesulfonyl)-N-methyl-L-tyrosyl]-4-phenylpipera zine(KN-62) (at 10 nM), inhibits rP2X7R activation by blocking BzATP-induced currents. However, they demonstrate less efficacy in inhibiting hP2X7R activation (40). The piperazine antagonist KN-62 blocks CaM kinase II, and experiments show inhibited ionic currents in KN-62-treated cells expressing hP2X7R or mP2X7R (206). The fourth group mainly consists of naturally synthesized compounds. Chelerythrine, among the benzophenanthridine alkaloids tested, is the only one that can effectively inhibit P2X7R function noncompetitively at ATP concentrations ranging from 0 to 1,000 μM (207). Mineral oil can also inhibit P2X7R function by reducing P2X7-dependent multinucleated giant cell formation, thus downregulating P2X7R expression (208). The fifth group comprises novel synthetic compounds with varied chemical structures and conformation types, including GSK-1482160, GSK-314181A, AZ1060612, AXX71, JNJ-54175446, and JNJ-55308942. An example of one such compound is 11C-GSK1482160, which exhibited high affinity and good binding-dissociation kinetics for P2X7R, as demonstrated by 11C-GSK1482160 *in vivo* PET/CT tracer kinetics in the experimenter (209). V-T exhibited the expected trend in lipopolysaccharide-treated mice (210). The results confirm the potential use of 11C-GSK1482160 as a novel radioligand for targeting P2X7R and as a biomarker of neuroinflammation, as evidenced by the activation of microglial cells via peripheral lipopolysaccharide treatment and the receptor-dependent regional binding (211). Interestingly, the specific P2X7 antagonist AZ10606120 completely blocked the ATP-induced release of SOD1 from NSC-34 cells (212). In contrast, the SOD1-G93A protein has recently been shown to be rapidly released into the extracellular space upon P2X7 activation and is then re-uptaken by naïve NSC-34 cells or microglia cell lines to induce endoplasmic reticulum stress and TNF-α release, which mediate neurodegenerative disease and neuroinflammation-related ALS events respectively (213). In the SOD1-G93A mouse model of ALS, AXX71 treatment resulted in a significant down-regulation of pro-inflammatory markers such as IL-1β, NOX2, and NF-κB in the spinal cord at the end of the disease. Additionally, AXX71 treatment regulated the levels of autophagy-related proteins LC3B-II and SQSTM1/p62 (199).

**Table 2:** P2X7R antagonists.

P2X7R antagonists	Type	BBB-penetrant	Efficacy	References
Puerarin	naturally synthesized compound	Yes	Inhibits sepsis mediated by NLRP3-Caspase-1-GSDMD and has neuroprotective properties in various neurological disorders such as HD, AD, epilepsy, cognitive disorders, anxiety disorders, and depression.	(214, 215)
Chelerythrine	naturally synthesized compound	Not found	Has a noncompetitive inhibitory action on the P2X7 receptor.	(207)
PPADS	nonselective antagonist	Yes	Prevents the deleterious effects of BzATP-treated microglia.	(216)
oxATP	ATP analogues	Yes	Almost complete blockade of ATP and BzATP induced currents reduces demyelination and ameliorates associated neurological symptoms.	(217)
BBG	nonselective antagonist	Yes	Reduces purinergic receptor expression levels, attenuates gliosis, reduces blood-brain barrier leakage, and prevents neuronal apoptosis.	(171, 204, 218)
calmidazolium	organic cations	Yes	Inhibition of BzATP-evoked currents and blockade of ion channel activation.	
A-438079	competitive antagonist	Yes	1. Prevents the 6-OHDA-induced depletion of striatal DA stores. 2.reduces electrographic and clinical seizure severity during status epilepticus and reduces seizure-induced neocortical neuronal death.	(70, 219)
KN-62	tyrosine derivative	Not found	Prevents excitotoxicity and loss of CCDPK II activity and Glu-induced reverse phosphorylation of endogenous proteins.	(220)
GSK-1482160	novel synthetic compound	Yes	Novel PET reagents as targeting P2X7 receptors.	(211)
GSK-314181A	novel synthetic compound	Yes	Has clinical utility as an anti-inflammatory and analgesic treatment.	(221)
JNJ-54175446	novel synthetic compound	Yes	Inhibits peripheral interleukin (IL)-1β release and attenuates dextroamphetamine-induced amelioration of mood and (visual) motor performance in a human dextroamphetamine-primed paradigm	(222)
JNJ-55308942	novel synthetic compound	Yes	Regulates IL-1β release and microglia activation	(223)
A-740003	novel synthetic compound	Yes	Inhibits reactive oxygen species (ROS) production and inhibits activation of the Nod-like receptor pyrin structural domain protein 3 (NLRP3) inflammatory vesicle and nuclear factor-κB (NF-κB) pathway.	(37, 224)
AXX71	novel synthetic compound	Yes	Effects on early symptoms of disease by reducing microglia-associated pro-inflammatory markers and autophagy.	(199)
CE-224, 535	selective antagonist	NO	The clinical candidate of rheumatoid arthritis.	(225)
Lu AF27139	selective antagonist	Yes	Diminishes colonic hypersensitivity and CNS prostanoid levels in a rat model of visceral pain.	(226)
PKT100	novel synthetic compound	Not found	Improves cardiac function and survival in pulmonary hypertension.	(227)

## Conclusion

The P2X7R is a non-selective ATP-gated cation channel that is widely expressed on the surface of various types of human cells. It plays an important role in the physiology and disease mechanisms of many human systems. For instance, the activation of P2X7R on skeletal muscle cells is involved in the pathogenesis of osteoarthritis (OA). Similarly, the activation of P2X7R in the cardiovascular system can induce small-vessel vasculitis, which may stimulate the development of hypertension and atherosclerosis. Additionally, ocular P2X7R may be associated with diseases of the retina. Finally, it has been demonstrated that P2X7R is inextricably involved in hematologic malignancies.

Due to its high-density localization in the nervous and immune systems, P2X7R plays a unique role in neuroinflammatory processes and is a significant factor in inducing various types of neurodegenerative diseases. When activated, P2X7R triggers the NLRP3 inflammasome, disrupting mitochondrial function and inducing Ca^2+^ influx into neurons. Elevated extracellular ATP levels resulting from neuronal apoptosis subsequently lead to ATP-induced oxidative stress and neurodegeneration, resulting in neuronal apoptosis and a significant degree of microglia activation and aggregation. Elevated extracellular ATP levels resulting from neuronal apoptosis subsequently lead to ATP-induced oxidative stress and neurodegeneration, resulting in neuronal apoptosis and a significant degree of microglia activation and aggregation. This, in turn, triggers a cascade of reactions by further activating P2X7R. Although microglia activation initially plays a neuroprotective role, as the disease progresses, it transitions from phagocytosis to the production of pro-inflammatory cytokines that worsen the disease. Activated microglia release excitotoxic glutamate, which can injure dopaminergic neurons.

Therefore, blocking the P2X7R pathway using a variety of approaches may be a practical and effective way to treat neurodegenerative disease and slow down its progression. This treatment has been shown to be effective in a number of neurodegenerative diseases. For instance, in AD, the P2X7R antagonist oxATP, as well as BBG and the Ca^2+^ efflux blocker Mg^2+^, have been shown to reduce A-β protein aggregation and subsequent plaque production. In HD, BBG has been found to prevent neuronal apoptosis and alleviate symptoms such as weight loss and motor coordination deficits. In ALS, the P2X7 antagonist AZ10606120 can reduce endoplasmic reticulum stress and the release of TNF-α, thereby counteracting the neuroinflammatory events associated with the disease. However, while antagonizing the P2X7R-mediated pathway shows promise for treating neurodegenerative diseases, it has not yet gained popularity in clinical settings. Therefore, the focus of major pharmaceutical laboratories studying P2X7 and neurodegenerative diseases is on refining and developing multiple forms of P2X7R antagonists and conducting clinical trials.

The relationship between P2X7R and microglia-specific phenotypic transformation is a controversial topic. Microglia are distinct from macrophages, as they have two cell types: pro-inflammatory phenotype (M1) and anti-inflammatory phenotype (M2), which express different markers on the cell membrane surface and play different roles. The former is involved in neurotoxicity, while the latter is associated with inflammation abatement and tissue repair. Environmental factors, such as LPS and IFN-γ, facilitate the conversion of microglia to the M1 phenotype. Conversely, IL-4 enables the M2 phenotype. Previous studies have shown that the activation of P2X7R by BzATP induces the polarization of the M1 phenotype, which promotes neuroinflammation. Other studies have found that astragalus polysaccharides, which are ATP-degrading agents, can enhance M2 polarization by reducing P2X7R activation. This exerts a protective effect on the nervous system. However, a related experiment found that P2X7R activation not only stimulates the formation of M1 markers but also promotes the production of M2 markers such as Arg-1(arginase-1). Controlling the activation of P2X7R by a certain amount of ATP may have neuroprotective effects. This finding is important for further research in this area.

## Author contributions

HZ: Writing – original draft. QL: Writing – original draft. SZ: Writing – original draft. HL: Writing – review & editing. WZ: Writing – review & editing.
